# Role of Commensal Microbes in the γ-Ray Irradiation-Induced Physiological Changes in *Drosophila melanogaster*

**DOI:** 10.3390/microorganisms9010031

**Published:** 2020-12-24

**Authors:** Hwa-Jin Lee, Shin-Hae Lee, Ji-Hyeon Lee, Yongjoong Kim, Ki Moon Seong, Young Woo Jin, Kyung-Jin Min

**Affiliations:** 1 Department of Biological Sciences, Inha University, Incheon 22212, Korea; dlghkwls1223@naver.com (H.-J.L.); lmjinee@gmail.com (S.-H.L.); 22171265@inha.edu (J.-H.L.); 2 Laboratory of Low Dose Risk Assessment, National Radiation Emergency Medical Center, Korea Institute of Radiological & Medical Sciences, Seoul 01812, Korea; wiseman@kirams.re.kr (Y.K.); skmhanul@kirams.re.kr (K.M.S.); jwjin@kirams.re.kr (Y.W.J.)

**Keywords:** γ-ray irradiation, commensal microbes, lifespan, fecundity, locomotion, mitochondria, reactive oxygen species (ROS), *Drosophila melanogaster*

## Abstract

Ionizing radiation induces biological/physiological changes and affects commensal microbes, but few studies have examined the relationship between the physiological changes induced by irradiation and commensal microbes. This study investigated the role of commensal microbes in the γ-ray irradiation-induced physiological changes in *Drosophila melanogaster*. The bacterial load was increased in 5 Gy irradiated flies, but irradiation decreased the number of operational taxonomic units. The mean lifespan of conventional flies showed no significant change by irradiation, whereas that of axenic flies was negatively correlated with the radiation dose. γ-Ray irradiation did not change the average number of eggs in both conventional and axenic flies. Locomotion of conventional flies was decreased after 5 Gy radiation exposure, whereas no significant change in locomotion activity was detected in axenic flies after irradiation. γ-Ray irradiation increased the generation of reactive oxygen species in both conventional and axenic flies, but the increase was higher in axenic flies. Similarly, the amounts of mitochondria were increased in irradiated axenic flies but not in conventional flies. These results suggest that axenic flies are more sensitive in their mitochondrial responses to radiation than conventional flies, and increased sensitivity leads to a reduced lifespan and other physiological changes in axenic flies.

## 1. Introduction

Currently, ionizing radiation is being used in various fields, such as cancer treatment [1] and sterilization [2]. Radiotherapy is a highly effective remedy that destroys cancer by subjecting the cancer cells to radiation. This method uses low or high linear energy radiation to kill tumor cells while minimizing the dose of radiation to healthy cells to prevent toxicity [3]. Sterilization by γ-ray irradiation is commonly used in many disciplines, such as drugs [2], healthcare products [4], and food packaging [5]. In addition to the benefits of radiation, there are also negative aspects of radiation. After the nuclear leak in Chernobyl [6] and Fukushima [7], people have had concerns about the adverse effects of radiation. Moreover, excessive doses of radioactive radon were recently detected in a bed mattress in Korea [8].

Several studies have examined the harmful effects of radiation on humans and animals. Next to smoking, radiation is the second highest cause of lung cancer [9], and ionizing radiation can have potentially damaging effects on the human brain [10]. In mammalian cells, ionizing radiation leads to lethal cell damage and chromosomal aberrations [11,12], and induces the downregulation of genes involved in the cell cycle regulation [13]. Radiation can also impair the locomotor activity and change the development time in experimental animal models, such as *Drosophila melanogaster* [14,15]. In addition, ionizing radiation has been reported to affect the proliferation of intestinal stem cells [16], alter the lifespan [17], and reduce fertility in *D. melanogaster* [18].

The pathological effects of ionizing radiation are associated mainly with the oxidization of subsequent damage to macromolecules, such as DNA [19]. Ionizing radiation releases electrons from atoms and molecules, generating ions that can break the covalent bonds of molecules, such as water and DNA. Ionizing radiation can also influence the DNA structure directly by breaking the DNA molecule [20]. Although the main target of ionizing radiation damage is considered to be the nucleus, recent reports have shown that mitochondria are the target organelles that can be damaged by ionizing radiation [19,21]. Ionizing radiation increases mitochondrial oxidative stress [22], affects the mitochondrial functions [23], and induces apoptosis. Radiation triggers mitochondria-encoded ATP synthase 6 gene expression changes related to cell survival [24]. In addition, ionizing radiation regulates the antioxidant response changes in vivo [25]. Most of the detrimental effects caused by radiation are oxidative stress-related damage. Oxidative stress is related to the generation of reactive oxygen species (ROS) via acute water radiolysis [19], as well as ROS occurrence and leakage from chronic mitochondrial damage [26]. The effects associated with mitochondrial impairment could result in aging-related changes and several diseases [27,28].

Ionizing radiation affects not only the host organism but also the commensal microbes that reside in the host’s body. Commensal microbes can regulate the health of the host by affecting the host’s development [29], immunity [30], and longevity [31], and these changes are dependent on the host’s physiology [32,33] and dietary factors [34,35,36]. Storelli et al. and Shin et al. demonstrated that altered microbes by undernutrition diet could regulate the growth and development of *D. melanogaster* through TOR and insulin signaling pathways [37,38]. In particular, a study using monocolonized mouse models showed that *Lactobacillus plantarum* promoted juvenile growth, similar to previous results in *D. melanogaster* [39].

Both ionizing and ultraviolet radiation can alter the abundance and composition of specific taxa within the microbiome [40,41]. For example, ultraviolet radiation has been shown to alter the diversity of the human skin microbiome [41]. Mice subjected to ionizing radiation exhibited a slight decrease in gut microbiota diversity compared with that of non-irradiated mice [40]. In addition, microbial alterations after irradiation in mice contributed to functional and metabolic shifts [40]. In cancer patients who received radiotherapy, the gut microbial composition was altered, with the number of species being dramatically reduced and the abundance also changing after radiotherapy [42]. In this regard, it is plausible that commensal microbes mediate the physiological changes resulting from radiation, but few studies have shown the roles of commensal microbes in the physiological changes after radiation exposure [43,44,45].

In this study, we used *D. melanogaster* as a model organism. The *Drosophila* microbiota is relatively simple, whereas that of vertebrates have complex diversity [46,47]. The dominant commensal bacteria resided in the intestine of *D. melanogaster* are *Acetobacteraceae*, *Lactobacillales,* and *γ-Proteobacteria* [46], but some species, such as *Enterococcus faecalis* [48], *Gluconobacter morbifer* [49], and *Enterobacteriaceae* [50], are also found in the *Drosophila* under laboratory-specific conditions.

Here, we examined whether the commensal microbiota can affect the physiological changes induced by γ-ray irradiation. Our results suggest that commensal microbes have a radioprotective effect on the lifespan, ROS generation, and mitochondrial changes induced by γ-ray irradiation.

## 2. Materials and Methods 

### 2.1. Fly Husbandry and Generation of Axenic (Axe) D. melanogaster

Oregon-R flies were used as the wild type. The flies were obtained from the Bloomington Stock Center (Indiana University, Bloomington, IN, USA) and had been adapting to the laboratory environment for the past 8 years. The flies were maintained at 25 °C and 70% relative humidity. The eggs were cultivated on sterile standard cornmeal–sugar–yeast (CSY, 5.2% cornmeal, 11% sugar, 2.5% instant yeast, 0.5% propionic acid, 0.04% methyl-4-hydroxybenzoate, and 1% agar) medium food bottles. After eclosion, the flies were maintained on sterile sugar–yeast (SY, 10% sugar, 10% instant yeast, 0.5% propionic acid, 0.04% methyl-4-hydroxybenzoate, and 1% agar) medium food vials under a 12 h light–dark cycle. For sterile media, each of the media mentioned above was autoclaved at 120 °C for 20 min, and all bottles or vials for food were exposed to ultraviolet (UV) light for 20 min on a clean bench. To exclude the possibility that the change of egg production of female flies by irradiation could influence the lifespan of female flies [51], all experiments with adults were performed using male flies except for the measurement of fecundity.

Axe flies were generated by bleaching the embryos, as described in a previous study [31]. The embryos were collected for 12 h and then dechorionated for 50 s in 5% sodium hypochlorite solution (Wako, Osaka, Japan), rinsed for 50 s in 70% ethanol, and washed for 1 min in sterile distilled water. The sterile embryos were transferred to sterile CSY medium bottles on a clean bench. Third-generation Axe flies from bleached eggs were used because the lifespan of Axe flies differs by the generation [31]. All conventional (Conv) and Axe adult flies were transferred to fresh sterile food every 2 days on a clean bench. The axenic conditions were confirmed by plating fly homogenate on plate count agar (PCA, Neogen Corporation, Lansing, MI, USA) containing 0.5% tryptone, 0.25% yeast extract, 0.1% glucose, and 1.5% bacto agar. 

### 2.2. γ-Ray Irradiation

Radiation tolerance varies greatly according to life stages in *D*. *melanogaster* [52]. We selected the third instar larval stage as the irradiation stage since it is more susceptible to radiation than the adult stage. The eggs were collected from 5- to 7-day-old adult female flies for 8 h on a sterile CSY medium. The feeding third instar larvae were subjected to radiation in a γ-ray irradiation machine at 0.1 Gy (dose rate of 0.67 cGy/min, 137Cs, MDI-KIRMAS 137, Seoul, Korea) or 5 Gy (dose rate of 3.25 Gy/min, 137Cs, Gammacell 3000 Elan, Nordion Inc., Ottawa, ON, Canada). After irradiation, the irradiated and non-irradiated larvae were transferred immediately to new sterile CSY media to exclude secondary effects arising from the microbes in media excreted by Conv flies. Non-irradiated and irradiated flies were maintained contemporaneously under the same conditions at 25 °C. 

### 2.3. Quantitative Analysis of Bacteria

The colony-forming units (CFUs) were determined according to the following procedure. The non-irradiated and irradiated adult flies were transferred to new SY food 1 day before the test. Five adult male flies (10 biological replicates) were collected in 1.5 mL tubes. To eliminate the bacteria on the fly surface, the flies were rinsed in 70% ethanol for 3 s and removed quickly. The flies were then homogenized with a tissue grinder-disposable plastic pellet pestle in sterile distilled water. The homogenates were diluted as necessary and plated onto MRS media (*Lactobacilli* MRS broth, BD & Difco, Sparks, MD, USA) or *Acetobacter*-selective (AS) media containing 2.5% D-mannitol (BD & Difco, Sparks, MD, USA), 0.5% yeast extract (BD & Difco, Sparks, MD, USA), 0.3% peptone (BD & Difco, Sparks, MD, USA), and 1.5% bacto agar. After plating the diluted homogenates, the media were cultivated for 2–3 days at 29 °C. The number of colonies was counted after colony formation. The data are presented as mean ± standard error of the mean (SEM) values.

For 16S rRNA PCR, the total genomic DNA from 30 adult male flies was extracted using a DNeasy Tissue Kit (Qiagen, Hilden, Germany) in accordance with the manufacturer’s instructions. The PCR assays were performed with a 60 °C annealing temperature and 40–60 cycles using taxon-specific 16S rRNA gene primers for the universal PCR primers (27F and 1492R). The sequences for the universal primer (27F, 1492R) were as follows: forward 5′-AGA GTT TGA TCM TGG CTC AG-3′, reverse 5′-TAC GGY TAC CTT GTT ACG ACT T-3’. After agarose gel electrophoresis, relative band intensity was quantified using ImageJ software (National Institutes of Health, Bethesda, MD, USA). Data are presented as mean ± SEM values.

### 2.4. Pyrosequencing of the 16S rRNA Gene

The dominant commensal microbe species in the gut of the irradiated and non-irradiated adult flies was determined by 454 pyrosequencing analysis of the 16S rRNA gene. Amplification, purification, and pyrosequencing of bacterial 16S rRNA gene sequences were performed at ChunLab Inc. (Seoul, Korea). The extracted genomic DNA from 150 guts dissected from surface-sterilized irradiated and non-irradiated adult male flies (5 biological replicates) was amplified using primers targeting the V3 to V4 hypervariable regions of the bacterial 16S rRNA gene. The sequences for the primer were as follows: V3-341F: 5′-X-AC-CCTACGGGNGGCWGCAG-3′, V4-805R: 5′-X-AC-GACTACHVGGGTATCTAATCC-3′, where X denotes a barcode uniquely designed for each sample followed by a common linker AC. The 16S rRNA gene amplicons were analyzed by pyrosequencing using the 454 GS FLX Titanium Sequencing System (Roche, Branford, CT, USA) at ChunLab Inc. 

To improve the data quality, low-quality (< Q25) reads were filtered using the Trimmomatic 0.32 read trimming tool. Among the quality-controlled raw data, paired-end sequence data (250 bp) were merged together using PANDAseq. The primers were trimmed with ChunLab’s in-house program at a similarity cut-off of 0.8. Nonspecific amplicons that do not encode 16S rRNA were detected by HMMER’s hmmsearch program with 16S rRNA profiles. The sequences were denoised using DUDE-Seq to correct for sequencing errors, and nonredundant reads were finally extracted using UCLUST clustering. The EzBioCloud 16S rRNA database was used for taxonomic assignment using USEARCH (8.1.1861_i86linux32), followed by more precise pairwise alignment UCHIME, and the nonchimeric 16S rRNA database from EzBioCloud was used to detect chimera on reads with <97% similarity. Reads that were not identified to the species level (with <97% similarity) in the EzBioCloud database were compiled, and UCLUST was used to perform de novo clustering to generate additional operational taxonomic units (OTUs). Pyrosequencing data were analyzed using the CLcommunity™ program, version 3.46. (ChunLab Inc., Seoul, Korea).

### 2.5. Lifespan Assay

Newly eclosed adult flies were collected over 48 h and then provided with a 1-day stabilizing period. The adult male flies were assigned randomly to sterile SY vials to a final density of 20 flies per vial (5 replicates). The vials were changed every 2 days for new vials containing fresh sterile SY media. During transfer, the dead flies were removed and recorded. Five replicate vials were established for each group, and the experiment was performed three times.

### 2.6. γH2AX Staining

To detect the double-strand breaks, the wing imaginal discs of the third instar larvae were dissected in ice-cold PBS and fixed for 1 h at room temperature in PBS containing 4% paraformaldehyde (Sigma-Aldrich, St. Louis, MO, USA). After washing and blocking for 1 h with PBS containing 0.1% Triton and 2% BSA, the samples were incubated at 4 °C overnight with anti-phospho-histone H2AX (γH2AX, Merck Millipore, Burlington, MA, USA). Subsequently, the samples were washed and incubated with the Cy3-conjugated goat anti-mouse secondary antibody (Jackson Immuno Research Laboratories, West Grove, PA, USA) for 2 h at room temperature. For visualization, the samples were mounted in VECTASHIELD mounting media (Vector Lab., Burlingame, CA, USA), and fluorescence images were acquired using a confocal microscope (LSM510 META, Carl Zeiss, Oberkochen, Germany). The ratio of γH2AX foci to nucleus area was quantified using ImageJ software. Data are presented as mean ± SEM values.

### 2.7. Measurement of Fecundity

Within the first 24 h of emergence, the virgin female flies were collected every 3 h and placed in SY food-containing vials at a fly density of one female and two males (10 replicates). Female virginity was confirmed by the absence of progeny in the food after 24 h. Each female was allotted 24 h for laying eggs. The flies were transferred to new sterile SY vials daily, and the number of eggs laid by each female was evaluated for 10 days. Ten vials were tested for each group. The data are presented as mean ± SEM values.

### 2.8. Measurement of Physical Activity

The locomotion performance was assessed by rapid iterative negative geotaxis (RING) assay. Newly eclosed adult flies were collected over 48 h, allowing a 1-day stabilization period. Male flies were assigned randomly to sterile SY vials to a final density of 10 flies per vial before performing a vertical climbing assay (13 replicates). Ten flies were loaded into the vertical climbing assay apparatus, which was then tapped on a tabletop three times in rapid succession to initiate a negative geotaxis response. The positions of the flies in the apparatus tubes were captured by obtaining digital images 4 s after the initiation of climbing behavior. The flies were assessed in consecutive trials separated by a minute of rest period. Thirteen replicates for each group were used in all the experiments for 5 weeks. The flies were transferred to new sterile SY vials three times a week. The data are presented as mean ± SEM values.

### 2.9. ROS Detection

The ROS in the gut was detected using DCFDA assays as described previously [53]. The guts from irradiated larvae or 7-day-old adult male flies were dissected in PBS and incubated in 40 μM CM-H2DCFDA [5-(and-6)-chloromethyl-2′,7′-dichloro-dihydrofluorescein diacetate, acetyl ester, Invitrogen, Waltham, MA, USA] for 10 min in the dark. After washing, the samples were mounted in VECTASHIELD mounting media, and fluorescence images were obtained using an inverted microscope (IX71, Olympus, Tokyo, Japan) equipped with a U-RFL-T mercury lamp (Olympus, Tokyo, Japan) at an excitation wavelength of 488 nm. Quantification of fluorescence intensity was conducted by ImageJ software. Data are presented as mean ± SEM values.

### 2.10. MitoTracker Red Staining

To detect the mitochondria in flies, the fat bodies of larvae or 7-day-old adult male flies were dissected in ice-cold PBS and fixed for 20 min at room temperature in PBS containing 4% paraformaldehyde. After washing and blocking with PBS containing 0.1% Triton and 2% BSA, the fat bodies were incubated with 500 nM MitoTracker red (Invitrogen, Waltham, MA, USA) for 30 min. The samples were mounted in VECTASHIELD mounting media, and fluorescence images were acquired using a confocal microscope.

### 2.11. Measurement of Mitochondrial DNA

The total genomic DNA from 30 adult male flies was extracted using a DNeasy Tissue Kit according to the manufacturer’s instructions (3 replicates). The mitochondrial DNA was quantified relative to nuclear DNA by the ratio of amplicons of cytochrome oxidase subunit I (*COI*) to amplicons of glyceraldehyde 3- phosphate dehydrogenase (*GAPDH*) in quantitative real-time PCRs. Quantitative PCR (qPCR) was performed using the QuantStudio™ 1 Real-Time PCR System (Applied Biosystems, Foster City, CA, USA). At least three replicates were established in each group. The data are presented as mean ± SEM. The qPCR assays were performed with a 60 °C annealing temperature and at 40 cycles. The sequences for the *COI* and *GAPDH* were as follows: *COI* forward 5′-GAA TTA GGA CAT CCT GGA GC-3′, *COI* reverse 5′-GCA CTA ATC AAT TTC CAA ATC C-3′, *GAPDH* forward 5′-GAC GAA ATC AAG GCT AAG GTC G-3′, *GAPDH* reverse 5′-AAT GGG TGT CGC TGA AGA AGT C-3′ [54].

### 2.12. Statistical Analysis

Log-rank tests were carried out to determine the statistical significance of differences in the results of survival analysis. The JMP statistical package (SAS, Cary, NC, USA) was used for the analyses. Spearman’s correlation coefficients, as provided in R 3.5.1 software, were used to analyze the correlation between the radiation dose and mean lifespan. The two-way analysis of variance (ANOVA), repeated measures ANOVA, and Tukey’s honestly significant difference (HSD) post hoc test using SPSS Statistical 21 (IBM, Armonk, NY, USA) were performed to assess the mean lifespan, fecundity, relative band intensity, CFU, operational taxonomic unit (OTU) numbers, and locomotion performance. A Student’s *t*-test was performed to compare the fluorescence intensity and the expression of mtDNA using the Excel Statistics Tool (Microsoft Corporation, Redmond, WA, USA).

## 3. Results

### 3.1. γ-Ray Irradiation Changes Commensal Microbe Flora in D. melanogaster

**Figure 1:**
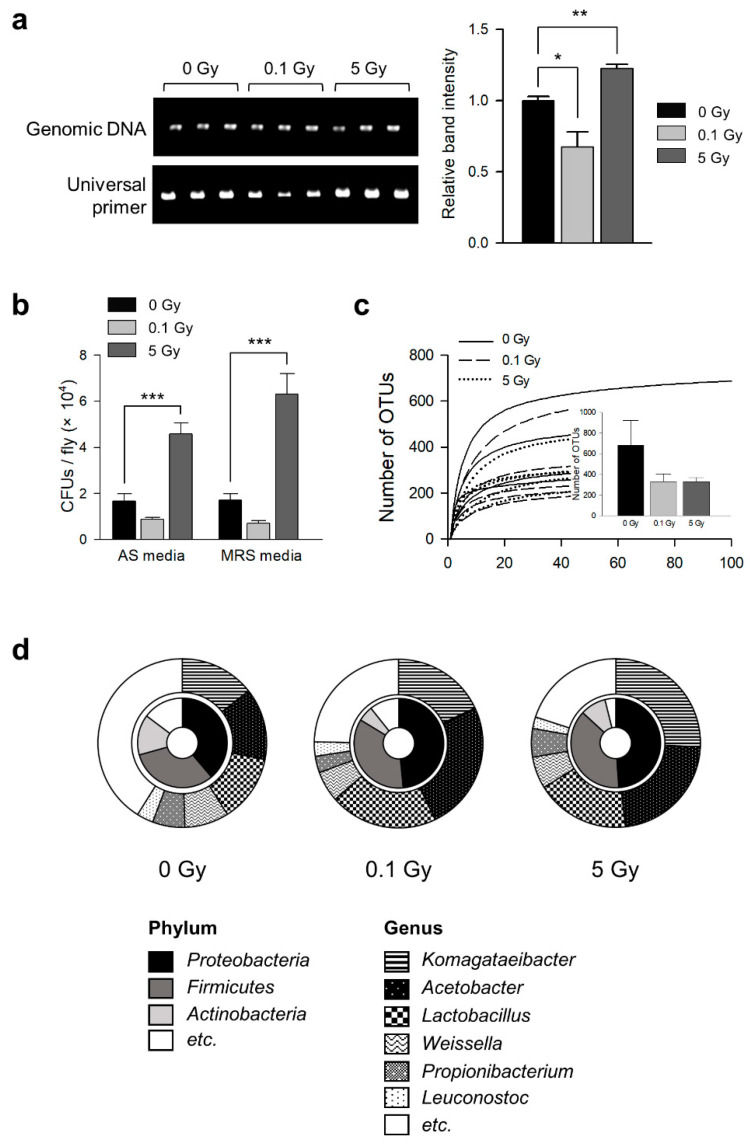
Microbial floral changes in *D*. *melanogaster* induced by γ-ray irradiation. (**a**) PCR assay of microbial 16S rRNA amplified gene using a universal primer (27F, 1492R). Microbial 16S rRNA gene sequences were amplified from the genomic DNA extracted from irradiated flies. ANOVA with Tukey’s post hoc test, * *p* < 0.05, ** *p* < 0.005. (**b**) The total number of CFUs from irradiated flies in *Acetobacter*-selective (AS) or MRS media plates. ANOVA with Tukey’s post hoc test, *** *p* < 0.001. (**c**) Rarefaction curves and the assigned number of operational taxonomic units (OTUs) obtained from the 454 pyrosequencing data. The inset shows the average number of OTUs in each group. The error bars represent the SEM. (**d**) Double pie charts of the bacterial compositions of irradiated flies. These charts show the major phylum and genus identified by 454 pyrosequencing of the 16S rRNA gene.

The effects of γ-ray irradiation on the commensal microbial flora in *D. melanogaster* were examined by measuring the changes in the microbial load and composition in 7-day-old *D. melanogaster* after 0.1 or 5 Gy irradiation at the third instar larval stage. In a previous report, 0.1 and 5 Gy were considered to be low and high doses of radiation, respectively [55,56]. Both the 16S rRNA PCR and CFU results showed that the abundance of commensal microbes was altered by irradiation (Figure 1). In the 16S rRNA PCR test, the bacterial load of the 5 Gy irradiated flies was increased compared with that of non-irradiated flies, but that of the 0.1 Gy irradiated flies was decreased (Figure 1a, ANOVA: F_2,6_ = 18.462, *p* = 0.003; Tukey’s HSD test: 0 vs. 0.1 Gy, *p* = 0.04, 0 vs. 5 Gy, *p* = 0.004). In the CFU test, the bacterial load of the 5 Gy irradiated flies was increased, but that of the 0.1 Gy irradiated flies was not changed in both the MRS and AS media (Figure 1b, AS media, ANOVA: F_2,27_ = 32.052, *p* < 0.001; Tukey’s HSD test: 0 vs. 0.1 Gy, *p* = 0.253, 0 vs. 5 Gy, *p* < 0.001; MRS media, ANOVA: F_2,27_ = 30.416, *p* < 0.001; Tukey’s HSD test: 0 vs. 0.1 Gy, *p* = 0.402, 0 vs. 5 Gy, *p* < 0.001).

This study examined whether the composition of microbial flora had been altered by γ-ray exposure through 454 pyrosequencing analysis of the 16S rRNA gene. There were 329 OTUs in both the 0.1 and 5 Gy irradiated flies, whereas 677 OTUs were detected in the non-irradiated flies (Figure 1c), indicating that the microbial species in the gut flora of *D. melanogaster* were decreased after γ-ray exposure. At the phylum level, *Proteobacteria* (including *Acetobacter* and *Komagataeibacter*), *Firmicutes* (including *Lactobacillus*, *Weissella*, and *Leuconostoc*), and *Actinobacteria* (including *Propionibacterium*) composed >80% of the microbiome in *D. melanogaster* (Figure 1d). At the genus level, *Komagataeibacter* (14.37% of the microbiome), *Acetobacter* (13.77%), *Lactobacillus* (12.89%), *Weissella* (8.49%), *Propionibacterium* (6.19%), and *Leuconostoc* (3.19%) were detected in the non-irradiated flies (Figure 1d). Comparatively, the proportions of *Acetobacter* (0.1 Gy 24.81%; 5 Gy 22.47%), *Lactobacillus* (0.1 Gy 20.66%; 5 Gy 25.73%), and *Komagataeibacter* (0.1 Gy 18.06%; 5 Gy 17.98%) were increased by γ-ray irradiation, but the proportions of *Weissella* (0.1 Gy 5.91%; 5 Gy 6.12%), *Propionibacterium* (0.1 Gy 3.15%; 5 Gy 5.48%), and *Leuconostoc* (0.1 Gy 2.75%; 5 Gy 2.22%) were decreased by γ-ray irradiation (Figure 1d). In addition, the bacterial diversity analyzed by the Shannon and Simpson diversity index was significantly reduced by γ-ray irradiation (Table 1). These results suggested that γ-ray irradiation at 0.1 and 5 Gy can decrease the diversity of the gut microbial flora and alter the gut microbial composition in *D. melanogaster*.

### 3.2. Effects of γ-Ray Irradiation on Lifespan and DNA Damage Response in Conventional and Axenic D. melanogaster

**Figure 2:**
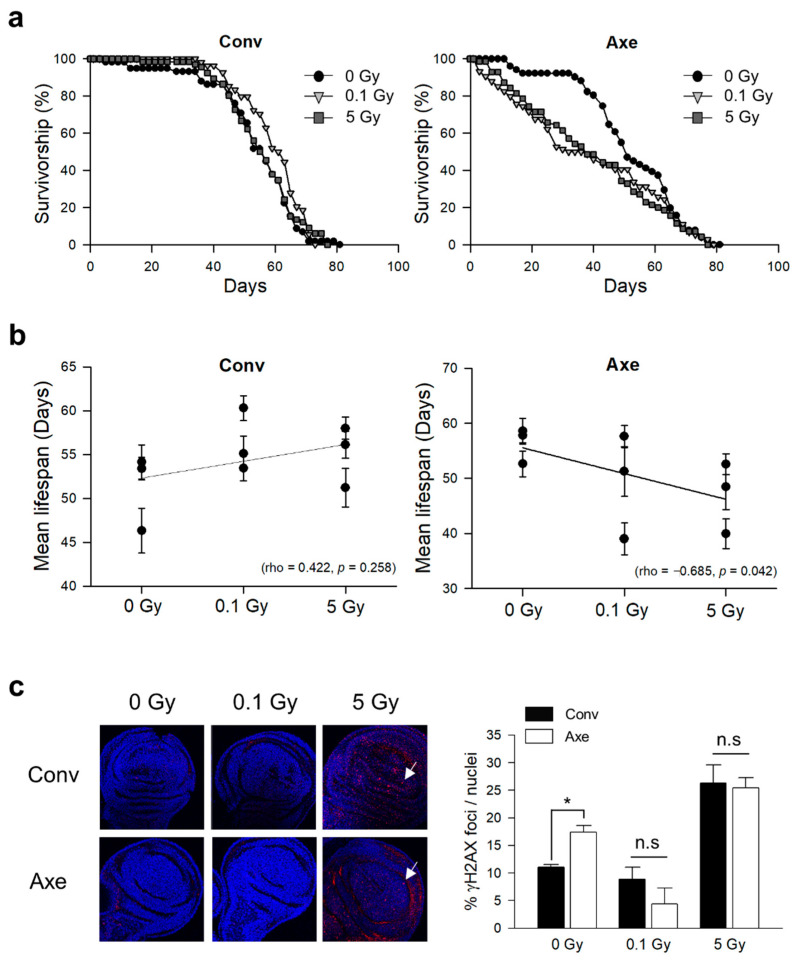
Effects of commensal microbes on the lifespan and DNA damage response of *D*. *melanogaster* after γ-ray irradiation. (**a**) Representative survival of the conventional (Conv) and axenic (Axe) flies after γ-ray irradiation at the third instar larval stage. (**b**) Mean lifespan of the Conv (Spearman’s correlation, rho = 0.422, *p* = 0.258) and Axe (Spearman’s correlation, rho = −0.685, *p* = 0.042) flies according to the radiation dose. The error bars represent the SEM. (**c**) The formation of radiation-induced γH2AX foci on wing imaginal discs of flies after γ-ray irradiation. Phosphorylated H2AX was used as a marker of DNA double-strand breaks. Foci on the imaginal discs of the Conv and Axe third instar larvae were detected by immunostaining with the specific antibodies for γH2AX. The arrows indicate the foci of DNA double-strand breaks on the wing imaginal discs of *D*. *melanogaster*. DAPI was used to stain the nuclei (blue). Original magnification was 400×. The graph represents the ratio of detected area of γH2AX foci to nuclei based on confocal microscopy images. Student’s *t*-test, * *p* < 0.05. n.s, not significant. The error bars represent the SEM.

The role of the commensal microbes on γ-ray irradiation-induced lifespan changes in *D. melanogaster* was assessed by examining the lifespan of conventional (Conv) and axenic (Axe) flies after 0.1 or 5 Gy γ-ray irradiation at the third instar larval stage. In all three trials, the lifespan of the Conv flies tended to increase after 0.1 or 5 Gy radiation exposure compared with 0 Gy radiation exposure (Figure 2a and Table 2). Interestingly, the lifespan of the Axe flies decreased after 0.1 or 5 Gy radiation exposure compared with that of 0 Gy radiation exposure (Figure 2a and Table 1). Spearman’s correlation analysis showed that the mean lifespan of the Conv flies was not correlated significantly with the radiation dose (Figure 2b, Spearman’s correlation, rho = 0.422, *p* = 0.258). In contrast, the mean lifespan of the Axe flies was negatively correlated with the radiation dose (Figure 2b, Spearman’s correlation, rho = −0.685, *p* = 0.042). These results suggest that commensal microbes have a radioprotective effect on the host.

Several reports indicate that commensal microbes have a distinct impact on DNA damage [57]. The level of DNA damage in the imaginal discs of the flies after radiation exposure was next measured to determine whether the DNA damage response was different between the Conv and Axe flies. The γH2AX foci, an indicator of DNA damage, was increased after 5 Gy irradiation at the third larval stage in both the Conv and Axe flies (Figure 2c), indicating that the flies were exposed to a radiation dose sufficient to increase the level of DNA damage. On the other hand, there was no significant difference in the DNA damage response between the Conv and Axe flies, suggesting that commensal microbes are not involved in the DNA damage response, and the aforementioned radioprotective effect of the commensal microbes may not be due to the protective effect on DNA damage.

**Figure 3:**
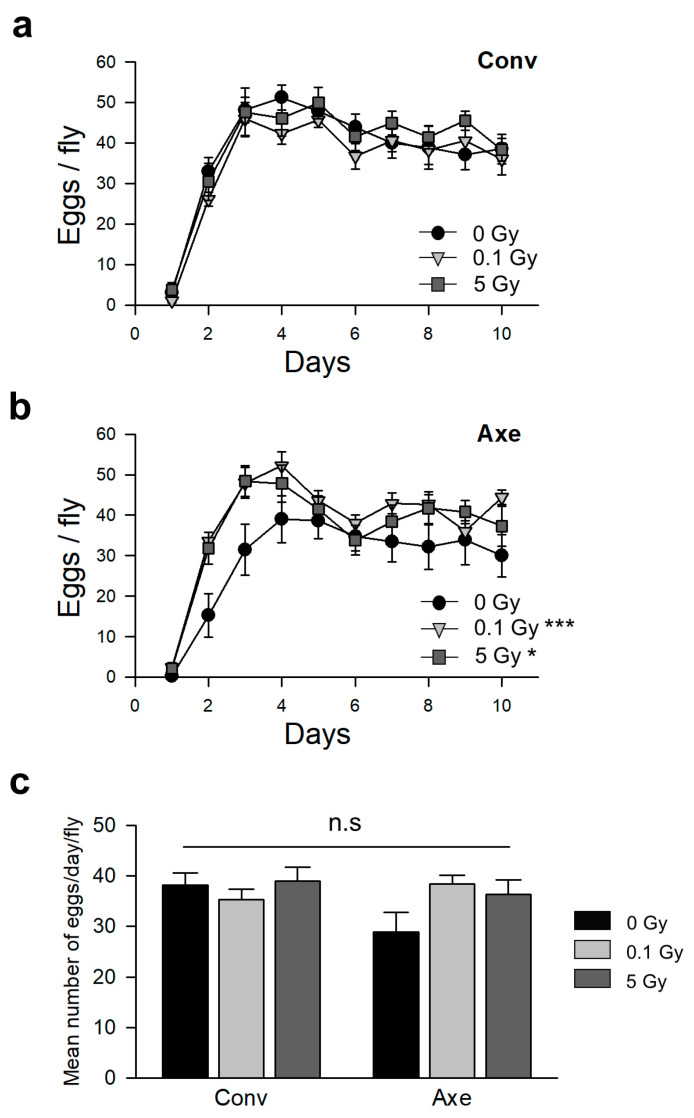
Fecundity of Conv or Axe *D. melanogaster* after γ-ray irradiation. The number of eggs per fly of the Conv (**a**) or Axe (**b**) flies for 10 days. The line with circles indicates the non-irradiated flies, the line with inverted triangles indicates the 0.1 Gy irradiated flies, and the line with squares indicates the 5 Gy irradiated flies. (**c**) The average number of eggs laid by the Conv or Axe flies after irradiation. The black bars indicate the average number of eggs laid by the non-irradiated flies, the light gray bars indicate the average number of eggs laid by the 0.1 Gy irradiated flies, and the dark gray bars indicate the average number of eggs laid by the 5 Gy irradiated flies. No significant (n.s) differences were detected between the groups. Two-way repeated measures ANOVA with Tukey’s post hoc test, * *p* < 0.05, *** *p* < 0.001. The error bars represent the SEM.

The effects of commensal microbes on the physiological changes resulting from irradiation were determined by measuring the fecundity and locomotion performance of the Conv and Axe flies after γ-ray irradiation. The daily number of eggs and the average number of eggs in the Conv flies did not change after γ-ray irradiation (Figure 3a,c). In contrast, the number of eggs of the Axe flies decreased compared with that of the Conv flies, but the decrease was not statistically significant (Figure 3c). γ-Ray irradiation increased the daily number of eggs in the Axe flies in some periods, but the average number of eggs was not significantly different after radiation (Figure 3b,c). These results suggest that neither radiation nor commensal microbes alter the fecundity.

**Figure 4:**
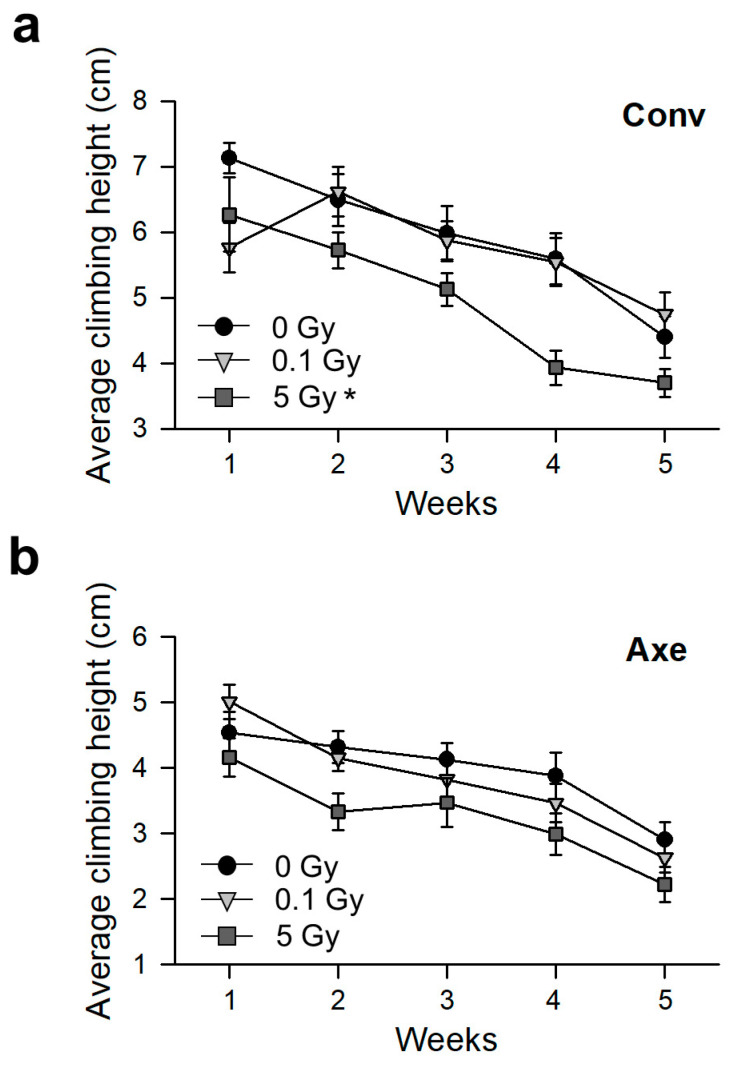
Effects of commensal microbes on the physical activity of Conv (**a**) or Axe (**b**) *D*. *melanogaster* after γ-ray irradiation. The physical activity was measured by assaying the vertical climbing behavior of the Conv or Axe flies after γ-ray irradiation. The line with circles indicates the non-irradiated flies, the line with inverted triangles indicates the 0.1 Gy irradiated flies, and the line with squares indicates the 5 Gy irradiated flies. Two-way repeated measures ANOVA with Tukey’s post hoc test, * *p* < 0.05. Error bars represent the SEM.

The vertical climbing activity of the Conv and Axe flies after radiation exposure was next measured. The climbing activity of the Conv flies was decreased by aging (Figure 4a, repeated measures ANOVA: F_4,60_ = 15.741, *p* < 0.001). The climbing activity of the Conv flies was also decreased by 5 Gy radiation exposure (Figure 4a, two-way repeated measures ANOVA: F_2,36_ = 3.975, *p* = 0.028; Tukey’s HSD test: 0 vs. 0.1 Gy, *p* = 0.833, 0 vs. 5 Gy, *p* = 0.029). Similar to the result in a previous report [58], the physical activity of the Axe flies was decreased compared with that of the Conv flies (Figure 4b). The climbing activity of the Axe flies was decreased by aging (Figure 4b, repeated measures ANOVA: F_4,60_ = 9.068, *p* < 0.001) but not by γ-ray irradiation (Figure 4b, two-way repeated measures ANOVA: F_2,36_ = 2.795, *p* = 0.074; Tukey’s HSD test: 0 vs. 0.1 Gy, *p* = 0.905, 0 vs. 5 Gy, *p* = 0.080). These results suggest that the commensal microbes are involved in the functional decline by radiation exposure.

### 3.3. Effects of Commensal Microbes on ROS Generation by γ-Ray Irradiation

Ionizing radiation generates ROS, which are associated with radiation-induced cytotoxicity resulting from oxidation of and damage to macromolecules, such as DNA and RNA [59]. The gut epithelia in contact with enteric commensal bacteria generate ROS rapidly to eliminate the excess bacteria proliferation and express several antioxidant systems to maintain the redox balance [60]. To determine whether the commensal microbes play a role in regulating oxidative stress after γ-ray irradiation, the guts of the Conv and Axe flies were stained with DCFDA, a ROS indicator, after 5 Gy irradiation. 

**Figure 5:**
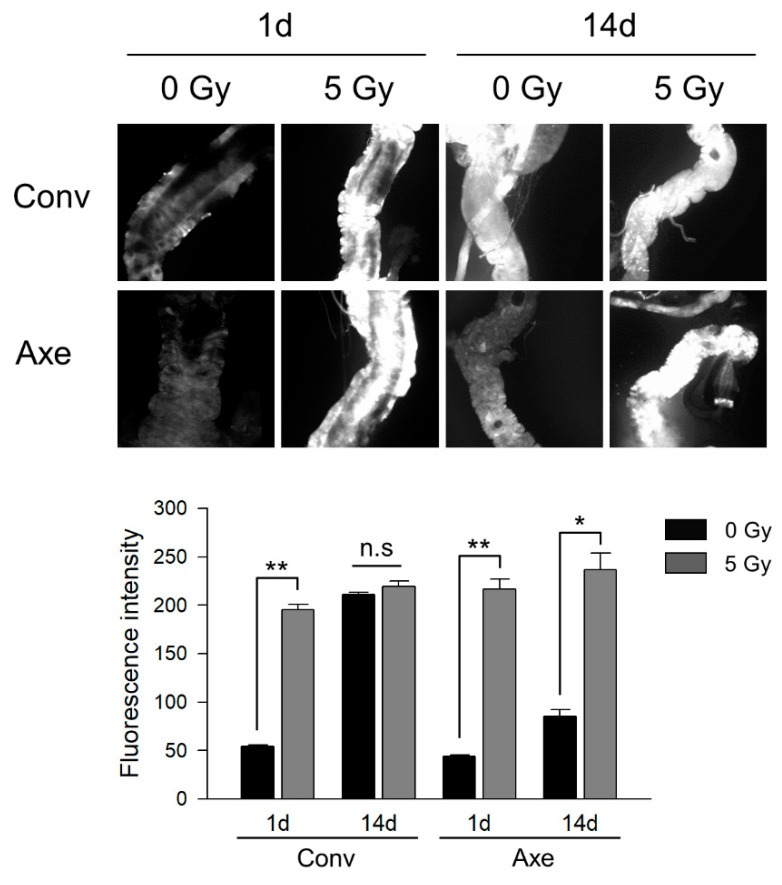
Effects of commensal microbes on ROS occurrence in the gut of *D*. *melanogaster* after γ-ray irradiation. ROS detection with DCFDA in the gut of the Conv or Axe flies at 1 and 14 days after γ-ray irradiation. Original magnification was 100×. The graph represents the fluorescence intensity based on microscopy images. Student’s *t*-test, * *p* < 0.05, ** *p* < 0.005. n.s, not significant. The error bars represent the SEM.

One day after irradiation, the fluorescence density increased in the Conv and Axe flies (Figure 5, second panel, *t*-test, Conv *p* = 0.001, Axe *p* = 0.004). At 14 days after irradiation, the density of fluorescence was greater in the non-irradiated Conv flies than in the non-irradiated Axe flies (Figure 5, third panel, *t*-test, *p* = 0.003), supporting the previous result regarding the role of commensal bacteria on ROS generation [61]. Irradiation did not further increase the fluorescence density in the Conv flies after 14 days, while the ROS signal increased in the Axe flies after 14 days (Figure 5, fourth panel, *t*-test, Conv *p* = 0.287, Axe *p* = 0.015). These results suggest that commensal microbes have a radioprotective role in ROS generation by irradiation, and intestinal microbes are related to the oxidative stress response following by γ-ray irradiation.

### 3.4. Effects of Commensal Microbes on Mitochondrial Change by γ-Ray Irradiation

**Figure 6:**
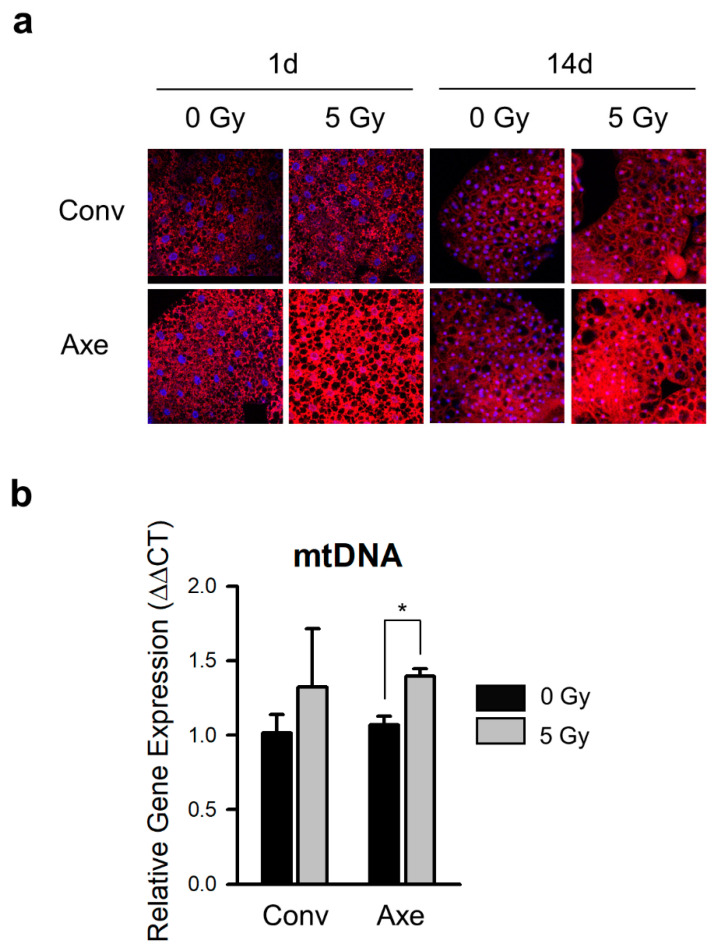
Effects of commensal microbes on the mitochondrial amount after γ-ray irradiation. (**a**) MitoTracker red-labeled mitochondria in the abdominal fat body of the Conv or Axe flies at 1 and 14 days after γ-ray irradiation. DAPI was used to stain the nuclei (blue). The original magnification was 400×. (**b**) The amount of mitochondrial DNA (mtDNA) relative to the nuclear DNA of the Conv or Axe flies was analyzed by examining the ratio of amplicons of COI to amplicons of GAPDH after the γ-ray irradiation. Student’s *t*-test, * *p* < 0.05 (*t*-test, Conv *p* = 0.496, Axe *p* = 0.014). The error bars represent the SEM.

Although ROS are produced by water radiolysis in irradiated cells, there is increasing evidence suggesting that the mitochondrial dysfunction induced by γ-irradiation is the leading cause of oxidative stress in irradiated cells, particularly in the long-term radiation effects [19,62]. In particular, radiation-induced mitochondrial dysfunction decreases the mitochondrial transmembrane potential and increases the mitochondrial mass [63,64]. To determine whether commensal microbes modulate radiation-induced ROS generation mediated by the mitochondrial metabolism, the mitochondria were labeled with MitoTracker red at 1 or 14 days after γ-ray irradiation. The amounts of labeled mitochondria in the Conv flies at 1 and 14 days were not altered significantly by γ-ray irradiation (Figure 6a, upper panel). In contrast, the signal of the labeled mitochondria was increased markedly after γ-ray irradiation in the Axe flies (Figure 6a, lower panel).

The mitochondria were quantified by measuring the amount of mitochondrial DNA (mtDNA) relative to nuclear DNA. Consistent with the results from the MitoTracker labeling, the amount of mtDNA was not altered significantly by γ-ray irradiation in the Conv flies. In contrast, it was increased by γ-ray irradiation in the Axe flies (Figure 6b, *t*-test, Conv *p* = 0.496, Axe *p* = 0.014). These results suggest that commensal microbes protect the host from the γ-ray irradiation-induced increase in mitochondrial quantity, highlighting the radioprotective effect of commensal microbes.

## 4. Discussion

Radiation can affect the lifespan of humans and experimental animals. Lifespan shortening was observed in humans exposed to high-dose irradiation in both Hiroshima and Nagasaki [65], and the lifespan of mice was reduced significantly by 0.5 Gy ionizing radiation [66]. Similarly, γ-irradiation has been reported to decrease the lifespan of fruit flies [56], and ultraviolet radiation has been shown to reduce the lifespan of nematodes [67]. Previously, it was well established that the absence of commensal microbes extends the lifespan in *Drosophila*, and the abundance of microbes is the major contributor in aging [31,68,69,70,71]. In addition, several studies have reported that the commensal microbiota is involved in the host health change following irradiation, but the studies are limited with no conclusive results. For example, Hou et al. reported that the treatment of broad-spectrum antibiotics to 6-week-old Kunming mice decreased their survival after total-body irradiation at a lethal dose of 12 Gy [45].

In contrast, Crawford and Gordon reported that total body irradiation of 16 Gy γ-ray to germ-free C57BL/6J mice produced fewer apoptotic cells in the small intestine compared with the control mice [44], indicating the role of commensal microbes on radiation-induced intestinal damage. Similarly, McLaughlin et al. reported that germ-free mice (ND-2) could better tolerate X-ray irradiation than the control mice [43]. Our study showed that commensal microbes have a radioprotective effect on lifespan shortening with γ-ray irradiation in *D. melanogaster*. Interestingly, γ-ray irradiation did not affect the lifespan of the Conv flies (Figure 2), which is different from previously reported results, showing that γ-ray irradiation decreased the lifespan of *D. melanogaster* significantly when flies were irradiated at the embryo stage [52]. This conflicting result can be interpreted as a difference in radiation tolerance between the embryo and third instar larvae. Feeding third instar larvae, the stage used in this study, have been reported to be a radioresistant life stage in *D. melanogaster* [52].

Radiation alters the gut microbiome, which is considered an important biomarker of host health [72]. Interestingly, the abundance and composition of specific taxa were reported to change after radiation exposure [40,41,73]. For example, the diversity of the gut microbiota was decreased in the fecal sample of mice subjected to ionizing radiation compared with that of non-irradiated mice [40]. In that study, there were significant perturbations on the relative abundances of bacteria in the order of *Bifidobacteriales*, *Coriobacteriales* (*Actinobacteria*), *Verrucomicrobiales* (*Verrucomicrobia*), and *Lactobacillales* (*Firmicutes*) after irradiation [40]. Similarly, in cancer patients receiving radiotherapy, the gut microbial composition was shown to be remodeled [42], with a dramatic reduction in the number of bacterial species. In particular, the abundances of *Firmicutes* and *Fusobacterium* were decreased significantly after radiation exposure [42]. Recently, Asimakis et al. reported that in melon flies, the application of irradiation to two distinct larval diets led to the formation of different bacterial profiles, including species richness, diversity, and composition. When melon flies were reared on an artificial bran-based diet, bacterial genera, such as *Raoultella* and *Citrobacter*, were reduced considerably, while sequences affiliated with members of *Providencia*, *Morganella*, and *Enterobacter* were increased. When flies were reared on sweet gourd, however, there was a significant decrease in species richness and minor differences in the relative abundance for members of *Enterobacter* and *Providencia* [73]. In the current study, the microbial floral change in *D. melanogaster* was analyzed after γ-ray irradiation at the third larval stage. γ-Ray irradiation increased the abundance of microbes at 5 Gy irradiation but decreased the diversity of microbial flora at both 0.1 and 5 Gy irradiation (Figure 1). The diversity of microbial flora is also a significant contributor to host health [74]. In particular, although our result is based on 16S rRNA sequencing, not full genome, two species identified with high specificity—*Acetobacter cerevisiae* and *Lactobacillus plantarum*—were increased after γ-ray exposure (*Acetobacter cerevisiae*, 0 Gy, 9.78%; 0.1 Gy, 20.3%; 5 Gy, 19.03%; *Lactobacillus plantarum*, 0 Gy, 4.34%; 0.1 Gy, 15.92%; 5 Gy, 20.89%). Although *Acetobacter cerevisiae* is one of the most frequently reported microbes in vinegar production [75,76], their role in the health benefit has not been reported. Several studies have reported that *Lactobacillus plantarum* could modulate the health by promoting systemic growth and inducing cellular ROS production in *Drosophila* [37,61].

Based on these results, several causes of the increases in specific species following γ-ray exposure are proposed. First, the predominant species that were increased by γ-ray exposure could be more radioresistant than the other species. *Lactobacillus sake* strains were reported to be resistant to γ-ray irradiation, with a 68% increase in resistance in the log phase over the stationary phase [77]. In addition, *Deinococcus radiodurans* has been reported to have 31 proteins that are upregulated by low-dose γ-ray irradiation, including proteins involved in DNA replication and repair [78]. Based on the expression of radiosensitive proteins, *Deinococcus radiodurans* has been described as being radioresistant. Therefore, investigating specific genes within a bacterium species could provide a novel perspective on the radiation defense systems. Second, an increased abundance of commensal bacteria could relate to the changes in immunity following γ-ray irradiation. Radiation influences the immune responses, including inflammation [55,79,80]. Stoecklein et al. reported that applying ionizing radiation to CD-1 mice produced chronic inflammasome activation in immune cells [81]. Moreover, ultraviolet radiation-induced immune suppression could alter the microbiome of the skin, suggesting that radiation-induced immune suppression could influence microbial proliferation [82]. Lastly, an environmental change associated with γ-ray exposure may be suitable for the proliferation of specific microbial species. After radiation exposure, changes in environmental factors, such as pH, oxygen concentration, and other growth-related elements, can result in a change in the composition of microbial flora [83]. Further studies will be needed to determine the radiation sensitivity of gnotobiotic flies inoculated with only *Lactobacillus plantarum* or *Acetobacter cerevisiae*.

In the early responses to ionizing radiation, radiolysis of water and activation of nitric oxide synthases (NOS) are the major sources of ROS in irradiated cells [19]. The mitochondria are the main source of ROS, consuming approximately 90% of the oxygen [84]. In the later stages of the response to radiation, the function of the mitochondria is disrupted, leading to delayed effects of radiation on oxidative stress. After X-ray radiation exposure of mouse heart tissue, there was a change in the mitochondrial oxidative metabolism [85]. In addition, when mouse NIH/3T3 cells were subjected to X-ray irradiation, the mtDNA level was increased in a time-dependent manner [64]. In this study, the ROS level after 5 Gy γ-ray irradiation was unaffected by the presence of commensal microbes at 1 day after irradiation (Figure 5). At 14 days after γ-ray irradiation, however, the ROS level was increased in the Axe flies, indicating that ROS generation in the later response to radiation is associated with the presence of commensal bacteria. In line with this finding, the change in the mitochondria quantity following γ-ray irradiation was suppressed by the presence of commensal microbes (Figure 6). The mitochondria–microbiota interaction has been actively investigated. The microbiota quality and diversity have been reported to modulate the mitochondria function. The metabolites released by microbiota modulate the mitochondrial respiratory chain and ATP production [86]. Moreover, a large quantity of hydrogen sulfide produced by several enteric bacteria inhibits the cytochrome respiratory chain activity in the colon [87], and nitric oxide (NO) produced by microbiota impairs the energy metabolism by decreasing acetyl-CoA production [88,89]. In addition, the microbiota-generated short-chain fatty acids (SCFAs) can be used as a donor of the mitochondrial electron transfer chain [90,91]. Although the effects of commensal microbes on the mitochondrial metabolism have been studied, there are few reports on the relationship between mitochondrial metabolism and commensal microbes in γ-ray irradiated conditions, and our results are novel findings in this respect.

## 5. Conclusions

In this study, γ-ray irradiation increased the bacterial abundance but decreased the diversity of the *Drosophila* microbiome. Although it is known that increased abundance of commensal microbes shortens the lifespan [31], irradiation did not cause significant change in the lifespan in conventional flies. In contrast, γ-ray irradiation shortened the lifespan of axenic flies. γ-Ray irradiation increased the ROS production and the amount of mitochondria in axenic flies compared with conventional flies. According to our results, commensal microbes have a radioprotective effect on the lifespan shortening and ROS generation induced by γ-ray irradiation. In line with the current trend of growing societal attention to radiation, this research provides a novel perspective that the presence of commensal microbes can mediate the physiological changes induced by radiation exposure. Our results showing the relationship of commensal microbes and physiological changes induced by γ-ray irradiation will provide fundamental knowledge to understand the underlying mechanisms of the somatic effects of ionizing radiation.

## Figures and Tables

**Table 1:** Summary for microbial diversity analysis by 454 pyrosequencing.

Group	OTU Number	Shannon	Simpson	Chao
0 Gy	677.40 ± 241.07	4.30 ± 0.54 ^a^	0.06 ± 0.02 ^a^	705.34 ± 241.91 ^a^
0.1 Gy	329.20 ± 76.18	2.94 ± 0.30 ^b^	0.16 ± 0.03 ^b^	340.07 ± 76.02 ^a^
5 Gy	329.40 ± 40.26	2.81 ± 0.40 ^b^	0.19 ± 0.06 ^b^	342.38 ± 42.11 ^a^

^a^ Values with different letters in a column indicate significant difference at *p* < 0.05 tested by one-way ANOVA with Tukey’s HSD test.

**Table 2:** Lifespan of the Conv and Axe flies after γ-ray irradiation at the third instar larval stage.

	Trial	Radiation Dose (Gy)	Mean Lifespan (Day)	Median Lifespan (Day)	Maximum Lifespan (Day)	Number of Flies	Log-Rank ^‡^		Wilcoxon ^‡^	
							ꭓ^2^	*p*-Value	ꭓ^2^	*p*-Value
Conv ^†^	1st	0	53.38 ± 1.29	57	61	140				
		0.1	53.44 ± 1.45	59	65	154	1.5165	0.2181	0.4455	0.5045
		5	58 ± 1.25	61	67	130	8.3963	0.0038 *	8.2578	0.0041 *
	2nd	0	46.32 ± 2.53	55	62	85				
		0.1	55.13 ± 1.97	60	66	78	3.8521	0.0497 *	6.8168	0.009 *
		5	51.21 ± 2.21	57	64	71	0.2432	0.6219	0.9642	0.3261
	3rd	0	54.16 ± 1.95	57	63	69				
		0.1	60.3 ± 1.42	62	63	68	4.9049	0.0268 *	5.3073	0.0212 *
		5	56.12 ± 1.53	56	63	66	0.1918	0.6614	0.0317	0.8588
Axe ^†^	1st	0	57.79 ± 1.32	59	67	157				
		0.1	57.6 ± 1.99	61	69	73	0.0274	0.8686	0.0408	0.8400
		5	52.53 ± 1.89	57	67	120	1.266	0.2605	3.204	0.0735
	2nd	0	58.57 ± 2.31	60	72	70				
		0.1	51.24 ± 4.48	53	70	25	1.8119	0.1783	2.1248	0.1449
		5	48.48 ± 4.18	60	68	44	1.4508	0.2284	2.1308	0.1444
	3rd	0	52.61 ± 2.34	51	66	51				
		0.1	39.01 ± 2.88	35	63	74	2.5024	0.1137	8.1454	0.0043 *
		5	39.94 ± 2.66	38	57	70	4.2501	0.0392 *	9.4652	0.0021 *

^†^ Conv, conventional fly; Axe, axenic fly; ^‡^ Log-rank and Wilcoxon tests were performed as comparisons with the 0 Gy result. * Asterisks indicate significant differences from the 0 Gy result.3.3. Effects of Commensal Microbes on the Reproduction and Locomotion Performance after γ-Ray Irradiation.

## Data Availability

The data that support the findings of this study are available from the corresponding author upon reasonable request.

## References

[1] Burnette B., Weichselbaum R.R. (2013). Radiation as an immune modulator. Semin. Radiat. Oncol..

[2] Abuhanoglu G., Ozer A.Y. (2014). Radiation sterilization of new drug delivery systems. Interv. Med. Appl. Sci..

[3] Baskar R., Dai J., Wenlong N., Yeo R., Yeoh K.W. (2014). Biological response of cancer cells to radiation treatment. Front. Mol. Biosci..

[4] Bortolin E., De Angelis C., Quattrini M.C., Barlascini O., Fattibene P. (2019). Detection of Ionizing Radiation Treatment in Glass Used for Healthcare Products. Radiat. Prot. Dosim..

[5] Iuliano A., Nowacka M., Rybak K., Rzepna M. (2019). The effects of electron beam radiation on material properties and degradation of commercial PBAT/PLA blend. J. Appl. Polym. Sci..

[6] Williams D. (2002). Cancer after nuclear fallout: Lessons from the Chernobyl accident. Nat. Rev. Cancer.

[7] Kamiya K., Ozasa K., Akiba S., Niwa O., Kodama K., Takamura N., Zaharieva E.K., Kimura Y., Wakeford R. (2015). Long-term effects of radiation exposure on health. Lancet.

[8] Seo S., Ha W.H., Kang J.K., Lee D., Park S., Kwon T.E., Jin Y.W. (2019). Health effects of exposure to radon: Implications of the radon bed mattress incident in Korea. Epidemiol. Health.

[9] Lubin J.H., Boice J.D. (1997). Lung cancer risk from residential radon: Meta-analysis of eight epidemiologic studies. J. Natl. Cancer Inst..

[10] Klatt A., Salzmann E., Schneider L.J., Reifschneider A., Korneck M., Hermle P., Burkle A., Stoll D., Kadereit S. (2019). Toxicity of ionizing radiation (IR) in a human induced pluripotent stem cell (hiPSC)-derived 3D early neurodevelopmental model. Arch. Toxicol..

[11] Bedford J.S. (1991). Sublethal damage, potentially lethal damage, and chromosomal aberrations in mammalian cells exposed to ionizing radiations. Int. J. Radiat. Oncol. Biol. Phys..

[12] Natarajan A.T., Berni A., Marimuthu K.M., Palitti F. (2008). The type and yield of ionising radiation induced chromosomal aberrations depend on the efficiency of different DSB repair pathways in mammalian cells. Mutat. Res..

[13] Beck M., Rombouts C., Moreels M., Aerts A., Quintens R., Tabury K., Michaux A., Janssen A., Neefs M., Ernst E. (2014). Modulation of gene expression in endothelial cells in response to high LET nickel ion irradiation. Int. J. Mol. Med..

[14] Shameer P.M., Sowmithra K., Harini B.P., Chaubey R.C., Jha S.K., Shetty N.J. (2015). Does exposure of male *Drosophila melanogaster* to acute gamma radiation influence egg to adult development time and longevity of F-1-F-3 offspring?. Entomol. Sci..

[15] Sudmeier L.J., Howard S.P., Ganetzky B. (2015). A *Drosophila* model to investigate the neurotoxic side effects of radiation exposure. Dis. Model Mech..

[16] Pyo J.H., Park J.S., Na H.J., Jeon H.J., Lee S.H., Kim J.G., Park S.Y., Jin Y.W., Kim Y.S., Yoo M.A. (2014). Functional modification of *Drosophila* intestinal stem cells by ionizing radiation. Radiat. Res..

[17] Moskalev A.A., Plyusnina E.N., Shaposhnikov M.V. (2011). Radiation hormesis and radioadaptive response in *Drosophila melanogaster* flies with different genetic backgrounds: The role of cellular stress-resistance mechanisms. Biogerontology.

[18] Morciano P., Iorio R., Iovino D., Cipressa F., Esposito G., Porrazzo A., Satta L., Alesse E., Tabocchini M.A., Cenci G. (2018). Effects of reduced natural background radiation on *Drosophila melanogaster* growth and development as revealed by the FLYINGLOW program. J. Cell Physiol..

[19] Azzam E.I., Jay-Gerin J.P., Pain D. (2012). Ionizing radiation-induced metabolic oxidative stress and prolonged cell injury. Cancer Lett..

[20] Lomax M.E., Folkes L.K., O’Neill P. (2013). Biological consequences of radiation-induced DNA damage: Relevance to radiotherapy. Clin. Oncol. R Coll. Radiol..

[21] Azimzadeh O., Scherthan H., Sarioglu H., Barjaktarovic Z., Conrad M., Vogt A., Calzada-Wack J., Neff F., Aubele M., Buske C. (2011). Rapid proteomic remodeling of cardiac tissue caused by total body ionizing radiation. Proteomics.

[22] Graziewicz M.A., Day B.J., Copeland W.C. (2002). The mitochondrial DNA polymerase as a target of oxidative damage. Nucleic Acids Res..

[23] Pandey B.N., Gordon D.M., De Toledo S.M., Pain D., Azzam E.I. (2006). Normal human fibroblasts exposed to high- or low-dose ionizing radiation: Differential effects on mitochondrial protein import and membrane potential. Antioxid. Redox. Signal.

[24] Kulkarni R., Marples B., Balasubramaniam M., Thomas R.A., Tucker J.D. (2010). Mitochondrial gene expression changes in normal and mitochondrial mutant cells after exposure to ionizing radiation. Radiat. Res..

[25] Lin R.X., Zhao H.B., Li C.R., Sun Y.N., Qian X.H., Wang S.Q. (2009). Proteomic analysis of ionizing radiation-induced proteins at the subcellular level. J. Proteome Res..

[26] Shimura T., Sasatani M., Kawai H., Kamiya K., Kobayashi J., Komatsu K., Kunugita N. (2017). A comparison of radiation-induced mitochondrial damage between neural progenitor stem cells and differentiated cells. Cell Cycle.

[27] DiMauro S., Hirano M. (2009). Pathogenesis and treatment of mitochondrial disorders. Adv. Exp. Med. Biol..

[28] Lane R.K., Hilsabeck T., Rea S.L. (2015). The role of mitochondrial dysfunction in age-related diseases. Biochim. Biophys. Acta.

[29] Keebaugh E.S., Yamada R., Obadia B., Ludington W.B., Ja W.W. (2018). Microbial Quantity Impacts *Drosophila* Nutrition, Development, and Lifespan. iScience.

[30] Ivanov I.I., Littman D.R. (2011). Modulation of immune homeostasis by commensal bacteria. Curr. Opin. Microbiol..

[31] Lee H.Y., Lee S.H., Lee J.H., Lee W.J., Min K.J. (2019). The role of commensal microbes in the lifespan of *Drosophila melanogaster*. Aging Albany N. Y..

[32] Han G., Lee H.J., Jeong S.E., Jeon C.O., Hyun S. (2017). Comparative Analysis of *Drosophila melanogaster* Gut Microbiota with Respect to Host Strain, Sex, and Age. Microb. Ecol..

[33] Ren C., Webster P., Finkel S.E., Tower J. (2007). Increased internal and external bacterial load during *Drosophila* aging without life-span trade-off. Cell Metab..

[34] Moschen A.R., Wieser V., Tilg H. (2012). Dietary Factors: Major Regulators of the Gut’s Microbiota. Gut Liver.

[35] Chaston J.M., Dobson A.J., Newell P.D., Douglas A.E. (2015). Host Genetic Control of the Microbiota Mediates the *Drosophila* Nutritional Phenotype. Appl. Environ. Microbiol..

[36] Dalby M.J., Ross A.W., Walker A.W., Morgan P.J. (2017). Dietary Uncoupling of Gut Microbiota and Energy Harvesting from Obesity and Glucose Tolerance in Mice. Cell Rep..

[37] Storelli G., Defaye A., Erkosar B., Hols P., Royet J., Leulier F. (2011). *Lactobacillus plantarum* Promotes *Drosophila* Systemic Growth by Modulating Hormonal Signals through TOR-Dependent Nutrient Sensing. Cell Metab..

[38] Shin S.C., Kim S.H., You H., Kim B., Kim A.C., Lee K.A., Yoon J.H., Ryu J.H., Lee W.J. (2011). *Drosophila* Microbiome Modulates Host Developmental and Metabolic Homeostasis via Insulin Signaling. Science.

[39] Schwarzer M., Makki M., Storelli G., Machuca-Gayet I., Srutkova D., Hermanova P., Martino M.E., Balmand S., Hudcovic T., Heddi A. (2016). *Lactobacillus plantarum* strain maintains growth of infant mice during chronic undernutrition. Science.

[40] Casero D., Gill K., Sridharan V., Koturbash I., Nelson G., Hauer-Jensen M., Boerma M., Braun J., Cheema A.K. (2017). Space-type radiation induces multimodal responses in the mouse gut microbiome and metabolome. Microbiome.

[41] Burns E.M., Ahmed H., Isedeh P.N., Kohli I., Van Der Pol W., Shaheen A., Muzaffar A.F., Al-Sadek C., Foy T.M., Abdelgawwad M.S. (2019). Ultraviolet radiation, both UVA and UVB, influences the composition of the skin microbiome. Exp. Dermatol..

[42] Nam Y.D., Kim H.J., Seo J.G., Kang S.W., Bae J.W. (2013). Impact of Pelvic Radiotherapy on Gut Microbiota of Gynecological Cancer Patients Revealed by Massive Pyrosequencing. PLoS ONE.

[43] McLaughlin M.M., Dacquisto M.P., Jacobus D.P., Horowitz R.E. (1964). Effects of the Germfree State on Responses of Mice to Whole-Body Irradiation. Radiat. Res..

[44] Crawford P.A., Gordon J.I. (2005). Microbial regulation of intestinal radiosensitivity. Proc. Natl. Acad. Sci. USA.

[45] Hou B., Xu Z.W., Zhang C.G. (2007). The effects of gut commensal bacteria depletion on mice exposed to acute lethal irradiation. J. Radiat. Res..

[46] Wong C.N., Ng P., Douglas A.E. (2011). Low-diversity bacterial community in the gut of the fruit fly *Drosophila melanogaster*. Environ. Microbiol..

[47] Ley R.E., Lozupone C.A., Hamady M., Knight R., Gordon J.I. (2008). Worlds within worlds: Evolution of the vertebrate gut microbiota. Nat. Rev. Microbiol..

[48] Cox C.R., Gilmore M.S. (2007). Native Microbial Colonization of *Drosophila melanogaster* and Its Use as a Model of *Enterococcus faecalis* Pathogenesis. Infect. Immun..

[49] Ryu J.H., Kim S.H., Lee H.Y., Bai J.Y., Nam Y.D., Bae J.W., Lee D.G., Shin S.C., Ha E.M., Lee W.J. (2008). Innate Immune Homeostasis by the Homeobox Gene Caudal and Commensal-Gut Mutualism in *Drosophila*. Science.

[50] Chandler J.A., Lang J.M., Bhatnagar S., Eisen J.A., Kopp A. (2011). Bacterial Communities of Diverse *Drosophila* Species: Ecological Context of a Host–Microbe Model System. PLoS Genet..

[51] Lamb M.J. (1964). The effects of radiation on the longevity of female *Drosophila subobscura*. J. Ins. Physiol..

[52] Paithankar J.G., Deeksha K., Patil R.K. (2017). Gamma radiation tolerance in different life stages of the fruit fly *Drosophila melanogaster*. Int. J. Radiat. Biol..

[53] Biosa A., Sanchez-Martinez A., Filograna R., Terriente-Felix A., Alam S.M., Beltramini M., Bubacco L., Bisaglia M., Whitworth A.J. (2018). Superoxide dismutating molecules rescue the toxic effects of PINK1 and parkin loss. Hum. Mol. Genet.

[54] Rera M., Bahadorani S., Cho J., Koehler C.L., Ulgherait M., Hur J.H., Ansari W.S., Lo T., Jones D.L., Walker D.W. (2011). Modulation of longevity and tissue homeostasis by the *Drosophila* PGC-1 homolog. Cell Metab..

[55] Seong K.M., Kim C.S., Lee B.S., Nam S.Y., Yang K.H., Kim J.Y., Park J.J., Min K.J., Jin Y.W. (2012). Low-dose radiation induces *Drosophila* innate immunity through Toll pathway activation. J. Radiat. Res..

[56] Seong K.M., Yu M., Lee K.S., Park S., Jin Y.W., Min K.J. (2015). Curcumin mitigates accelerated aging after irradiation in *Drosophila* by reducing oxidative stress. Biomed. Res. Int..

[57] Allen J., Sears C.L. (2019). Impact of the gut microbiome on the genome and epigenome of colon epithelial cells: Contributions to colorectal cancer development. Genome Med..

[58] Heys C., Lize A., Blow F., White L., Darby A., Lewis Z.J. (2018). The effect of gut microbiota elimination in *Drosophila melanogaster*: A how-to guide for host-microbiota studies. Ecol. Evol..

[59] Panganiban R.A., Snow A.L., Day R.M. (2013). Mechanisms of radiation toxicity in transformed and non-transformed cells. Int. J. Mol. Sci..

[60] Wentworth C.C., Alam A., Jones R.M., Nusrat A., Neish A.S. (2011). Enteric commensal bacteria induce extracellular signal-regulated kinase pathway signaling via formyl peptide receptor-dependent redox modulation of dual specific phosphatase 3. J. Biol. Chem..

[61] Jones R.M., Luo L., Ardita C.S., Richardson A.N., Kwon Y.M., Mercante J.W., Alam A., Gates C.L., Wu H., Swanson P.A. (2013). Symbiotic *lactobacilli* stimulate gut epithelial proliferation via Nox-mediated generation of reactive oxygen species. EMBO J..

[62] Datta K., Suman S., Kallakury B.V., Fornace A.J. (2012). Exposure to heavy ion radiation induces persistent oxidative stress in mouse intestine. PLoS ONE.

[63] Kim E.M., Yang H.S., Kang S.W., Ho J.N., Lee S.B., Um H.D. (2008). Amplification of the gamma-irradiation-induced cell death pathway by reactive oxygen species in human U937 cells. Cell Signal..

[64] Yamamori T., Sasagawa T., Ichii O., Hiyoshi M., Bo T., Yasui H., Kon Y., Inanami O. (2017). Analysis of the mechanism of radiation-induced upregulation of mitochondrial abundance in mouse fibroblasts. J. Radiat. Res..

[65] Jordan B.R. (2016). The Hiroshima/Nagasaki Survivor Studies: Discrepancies between Results and General Perception. Genetics.

[66] Dalke C., Neff F., Bains S.K., Bright S., Lord D., Reitmeir P., Rossler U., Samaga D., Unger K., Braselmann H. (2018). Lifetime study in mice after acute low-dose ionizing radiation: A multifactorial study with special focus on cataract risk. Radiat. Environ. Biophys..

[67] Prasanth M.I., Santoshram G.S., Bhaskar J.P., Balamurugan K. (2016). Ultraviolet-A triggers photoaging in model nematode *Caenorhabditis elegans* in a DAF-16 dependent pathway. Age Dordr..

[68] Li H., Qi Y., Jasper H. (2016). Preventing Age-Related Decline of Gut Compartmentalization Limits Microbiota Dysbiosis and Extends Lifespan. Cell Host Microbe..

[69] Iatsenko I., Boquete J.P., Lemaitre B. (2018). Microbiota-Derived Lactate Activates Production of Reactive Oxygen Species by the Intestinal NADPH Oxidase Nox and Shortens *Drosophila* Lifespan. Immunity.

[70] Clark R.I., Salazar A., Yamada R., Fitz-Gibbon S., Morselli M., Alcaraz J., Rana A., Rera M., Pellegrini M., Ja W.W. (2015). Distinct Shifts in Microbiota Composition during *Drosophila* Aging Impair Intestinal Function and Drive Mortality. Cell Rep..

[71] Guo L., Karpac J., Tran S.L., Jasper H. (2014). PGRP-SC2 promotes gut immune homeostasis to limit commensal dysbiosis and extend lifespan. Cell.

[72] McBurney M.I., Davis C., Fraser C.M., Schneeman B.O., Huttenhower C., Verbeke K., Walter J., Latulippe M.E. (2019). Establishing What Constitutes a Healthy Human Gut Microbiome: State of the Science, Regulatory Considerations, and Future Directions. J. Nutr..

[73] Asimakis E.D., Khan M., Stathopoulou P., Caceres C., Bourtzis K., Tsiamis G. (2019). The effect of diet and radiation on the bacterial symbiome of the melon fly, Zeugodacus cucurbitae (Coquillett). BMC Biotechnol..

[74] Clemente J.C., Ursell L.K., Parfrey L.W., Knight R. (2012). The impact of the gut microbiota on human health: An integrative view. Cell.

[75] Torija M.J., Mateo E., Guillamon J.M., Mas A. (2010). Identification and quantification of acetic acid bacteria in wine and vinegar by TaqMan-MGB probes. Food Microbiol..

[76] Gomes R.J., Borges M.F., Rosa M.F., Castro-Gomez R.J.H., Spinosa W.A. (2018). Acetic Acid Bacteria in the Food Industry: Systematics, Characteristics and Applications. Food Technol. Biotechnol..

[77] Hastings J.W., Holzapfel W.H., Niemand J.G. (1986). Radiation resistance of *lactobacilli* isolated from radurized meat relative to growth and environment. Appl. Environ. Microbiol..

[78] Lu H., Gao G., Xu G., Fan L., Yin L., Shen B., Hua Y. (2009). *Deinococcus radiodurans* PprI switches on DNA damage response and cellular survival networks after radiation damage. Mol. Cell Proteom..

[79] El-Saghire H., Thierens H., Monsieurs P., Michaux A., Vandevoorde C., Baatout S. (2013). Gene set enrichment analysis highlights different gene expression profiles in whole blood samples X-irradiated with low and high doses. Int. J. Radiat. Biol..

[80] Lumniczky K., Candeias S.M., Gaipl U.S., Frey B. (2017). Editorial: Radiation and the Immune System: Current Knowledge and Future Perspectives. Front Immunol..

[81] Stoecklein V.M., Osuka A., Ishikawa S., Lederer M.R., Wanke-Jellinek L., Lederer J.A. (2015). Radiation exposure induces inflammasome pathway activation in immune cells. J. Immunol..

[82] Patra V., Byrne S.N., Wolf P. (2016). The Skin Microbiome: Is It Affected by UV-induced Immune Suppression?. Front Microbiol..

[83] Hieken T.J., Chen J., Hoskin T.L., Walther-Antonio M., Johnson S., Ramaker S., Xiao J., Radisky D.C., Knutson K.L., Kalari K.R. (2016). The Microbiome of Aseptically Collected Human Breast Tissue in Benign and Malignant Disease. Sci. Rep..

[84] Li X., Fang P., Mai J., Choi E.T., Wang H., Yang X.F. (2013). Targeting mitochondrial reactive oxygen species as novel therapy for inflammatory diseases and cancers. J. Hematol. Oncol..

[85] Barjaktarovic Z., Schmaltz D., Shyla A., Azimzadeh O., Schulz S., Haagen J., Dorr W., Sarioglu H., Schafer A., Atkinson M.J. (2011). Radiation-induced signaling results in mitochondrial impairment in mouse heart at 4 weeks after exposure to X-rays. PLoS ONE.

[86] Clark A., Mach N. (2017). The Crosstalk between the Gut Microbiota and Mitochondria during Exercise. Front Physiol..

[87] Goubern M., Andriamihaja M., Nubel T., Blachier F., Bouillaud F. (2007). Sulfide, the first inorganic substrate for human cells. FASEB J..

[88] Leschelle X., Goubern M., Andriamihaja M., Blottiere H.M., Couplan E., Gonzalez-Barroso M.D., Petit C., Pagniez A., Chaumontet C., Mignotte B. (2005). Adaptative metabolic response of human colonic epithelial cells to the adverse effects of the luminal compound sulfide. Biochim. Biophys. Acta.

[89] Vermeiren J., Van de Wiele T., Van Nieuwenhuyse G., Boeckx P., Verstraete W., Boon N. (2012). Sulfide- and nitrite-dependent nitric oxide production in the intestinal tract. Microb. Biotechnol..

[90] Donohoe D.R., Garge N., Zhang X., Sun W., O’Connell T.M., Bunger M.K., Bultman S.J. (2011). The microbiome and butyrate regulate energy metabolism and autophagy in the mammalian colon. Cell Metab..

[91] Yadav H., Lee J.H., Lloyd J., Walter P., Rane S.G. (2013). Beneficial metabolic effects of a probiotic via butyrate-induced GLP-1 hormone secretion. J. Biol. Chem..

